# Long-term pulmonary function after posterior spinal fusion in main thoracic adolescent idiopathic scoliosis

**DOI:** 10.1371/journal.pone.0235123

**Published:** 2020-06-25

**Authors:** Young Man Byun, Takahiro Iida, Katsuhisa Yamada, Kuniyoshi Abumi, Terufumi Kokabu, Akira Iwata, Norimasa Iwasaki, Hideki Sudo

**Affiliations:** 1 Department of Orthopaedic Surgery, Hokkaido University Hospital, Sapporo, Hokkaido, Japan; 2 First Department of Orthopaedic Surgery, Dokkyo Medical University Saitama Medical Center, Koshigaya, Saitama, Japan; University of California San Francisco, UNITED STATES

## Abstract

**Background:**

Adolescent idiopathic scoliosis (AIS) patients typically undergo surgical treatment as teenagers, follow-ups of >5 years are necessary to evaluate effects on peak pulmonary reserves. However, limited data is available regarding the long-term (>10 years) effects of surgical intervention on pulmonary function (PF) in patients with thoracic AIS.

**Objective:**

To provide long-term (>10 years) information on the PF after posterior spinal fusion for treating main thoracic AIS. We especially investigated whether surgical correction for AIS led to impairment of the PF.

**Methods:**

A total of 35 patients with main thoracic AIS treated with posterior spinal fusion were included. Radiographs and PF tests, which included measurements of absolute and percent-predicted values of forced vital capacity (FVC) and forced expiratory volume in 1 second (FEV_1_), were evaluated.

**Results:**

Mean age at surgery was 14.9 years (12–19 years). Mean follow-up period was 15.1 years (10–24 years). Although the final postoperative FVC and FEV_1_ absolute values were higher than the preoperative values, the differences were not statistically significant (p = 0.22 and p = 0.08, respectively). Percent-predicted FVC and FEV_1_ values between preoperative and final postoperative measurements were not statistically different (p = 0.63 and p = 0.29, respectively). However, for the patients who presented with pulmonary impairment preoperatively, both the FVC and FEV_1_ significantly increased at the final follow-up (p = 0.01 and p = 0.01, respectively).

**Conclusions:**

Long-term results of AIS patients who underwent posterior spinal fusion in main thoracic curves demonstrated absolute and percent-predicted PF test values similar to preoperative measurements; thus, indicating that posterior spinal fusion did not decrease PF 15 years after the initial surgery. Instead, patients with severe preoperative pulmonary impairment might show some degree of improvement after surgery.

## Introduction

Posterior spinal fusion is widely applied for the treatment of adolescent idiopathic scoliosis (AIS) and can significantly ameliorate the spinal deformity. In addition to correction of the deformity, one of the other important indications for surgical correction of thoracic AIS may include prevention of pulmonary morbidity [1,2]. In a long-term follow-up study on untreated AIS patients, pulmonary function (PF) was affected in patients with thoracic curves and a clinically significant decrease in PF occurred in thoracic curves >100° [1].

While there were no significant differences in PF tests after anterior spinal fusion for thoracolumbar/lumbar (TL/L) AIS patients who were followed-up >10 years [3–5], some studies have documented that surgical correction of thoracic AIS can stabilise or improve PF if posterior spinal fusion is used; however, decreases in PF may be associated with the anterior approach in which a thoracotomy is performed in the surgical procedure [6–8]. These results suggest that the surgical approach is an important factor that can affect postoperative PF in patients with thoracic AIS.

Because AIS patients typically undergo surgical treatment as teenagers, follow-ups of >5 years are necessary to evaluate effects on peak pulmonary reserves [3, 5, 7, 8]. However, limited data is available regarding the long-term (>10 years) effects of surgical intervention on PF in patients with thoracic AIS [5, 7–10]. Especially, few studies have reported on long-term (>10 years) PF after posterior spinal fusion in patients with thoracic AIS [5, 9, 10]. In addition, major limitations of the studies were that less than 10% of patients returned for the long-term follow-up and few patients (7–18 patients) were included [5, 10]. Furthermore, all patients in one of the studies were treated by posterior spinal fusion with Harrington rod instrumentation [10]. Therefore, the purpose of this study was to analyse the effect of posterior spinal fusion on PF in AIS patients with main thoracic (MT) curves with a long-term follow-up >10 years. We especially investigated whether surgical correction for AIS led to impairment of the PF.

## Materials and methods

After institutional review board approval, data on a series of consecutive patients with AIS and MT curve (Lenke type 1–4) who underwent posterior correction at our institution between 1991 and 2005 were included and retrospectively reviewed. The Ethics Committee of Hokkaido University Hospital approved this research. Written consents were obtained from all the subjects, and when applicable from their guardians. With respect to the Lenke classification, type 1 is a single MT curve, type 2 is a double thoracic curve, type 3 is a MT-main TL/L double curve, type 4 is a triple major curve in the proximal thoracic (PT), MT, and TL/L regions, type 5 is a single TL/L curve, and type 6 is a TL/L-main thoracic double curve. Exclusion criteria included syndromic, neuro-muscular and congenital scoliosis, follow-up for <10 years, and presence of other TL/L scoliosis curves such as Lenke type 5 and type 6 curves. None of the patients included had clinical lung function issues. All patients underwent a posterior spinal fusion with segmental pedicle screw in a lumbar lesion and hook and segmental wiring in a thoracic lesion (hybrid construct) using ISOLA instrumentation system (DePuy AcroMed, Raynham, MA, USA) without thoracoplasty. Radiographs and PF tests were evaluated. The preoperative data were retrospectively reviewed, while the follow-up data were prospectively collected by spine surgeons independent from the treatment team. A total of 50 consecutive AIS patients with MT curve (Lenke type 1–4) underwent posterior correction during the period of study. Thirty-five patients (seven males, 28 females) were willing to participate and returned for a minimum 10-year follow-up.

Standing long-cassette posteroanterior and lateral radiographs were evaluated for multiple parameters before and after surgery and at the final follow-up. Coronal and sagittal Cobb measurements of the PT, MT, and TL/L curves were obtained. The end vertebrae levels were determined on preoperative radiographs and were measured on subsequent radiographs to maintain consistency for statistical comparisons [3, 7, 8, 11]. Sagittal measurements included thoracic kyphosis (T5–12) and lumbar lordosis (L1–S1). Coronal balance was measured by lateral displacement of the C7 coronal plumb line from the centre sacral vertical line (CSVL). Sagittal balance was evaluated by measuring the absolute displacement value of the C7 plumb line relative to the S1 posterior superior corner as the sagittal vertical axis.

Preoperative and final follow-up PF tests, which included measurements of the forced vital capacity (FVC) and forced expiratory volume in 1 second (FEV_1_), were conducted in the standing position. Each measurement was repeated thrice; the highest values of the absolute and percent-predicted values were used. The FVC is the vital capacity from a maximally forced expiratory effort, and the FEV_1_ is the volume expired at the end of the first second of forced expiration. Based on the American Thoracic Society’s guidelines, severity of pulmonary impairment were categorised as follows: no pulmonary impairment (percent-predicted FVC > 80%); mild impairment (65% < percent-predicted FVC ≤ 80%); moderate impairment (50% < percent-predicted FVC ≤ 65%); and severe impairment (percent-predicted FVC ≤ 50%) [4, 12]. In addition, airflow obstruction was assessed with the Global Initiative for Chronic Obstructive Lung Disease (GOLD) classification [13]. In obstructive lung disease, the FEV_1_ is reduced, resulting in a decrease in the FEV_1_/FVC ratio. An FEV_1_/FVC < 0.7 is the spirometric criterion for airflow limitation, and the limitation is further assessed based on the percent-predicted FEV_1_ as follows: GOLD 1 (percent-predicted FEV_1_ ≥ 80); GOLD 2 (50 < percent-predicted FEV_1_ < 79%); GOLD 3 (30 ≤ percent-predicted FEV_1_ ≤ 49); and GOLD 4 (percent-predicted FEV_1_ < 30) [13].

### Statistical analysis

All statistical computations were carried out using JMP Pro version 14.0 statistical software (SAS Institute, Cary, NC, USA). Comparisons of PF tests and radiographic quantitative variables were performed using a paired t-test or a Mann-Whitney *U* test. Pearson’s correlation coefficient analyses were used to assess the relationships between two continuous variables. Statistical significance was defined as p <0.05.

## Results

### Patient demographic data

Patient demographic data are summarised in Table 1. Mean age at the time of surgery was 14.9 years (12–19 years). The mean follow-up time was 15.1 years (10–24 years), and mean age at time of follow-up was 34.1 years (26–44 years). None of the patients had undergone re-operation. On an average, 10.9 segments (8–13 segments) were instrumented. The upper-most instrumented vertebra was T2 in 12 patients; T3, 12 patients; T4, 9 patients; and T5, 2 patients. The lowest instrumented vertebra was T12 in 2 patients; L1, 7 patients; L2, 8 patients; and L3, 18 patients.

**Table 1:** Patient demographic data.

	Mean ± standard deviation	Range
Age at surgery (yrs)	14.9 ± 2.0	12 to 19
Present age (yrs)	34.1 ± 4.8	26 to 44
Risser sign	3.8 ± 1.0	0 to 5
Cobb length (upper end to lower end vertebra)	9.3 ± 2.6	5 to 14
Instrumentation length (segments)	10.9 ± 1.5	8 to 13
Follow-up periods (yrs)	15.1 ± 4.4	10 to 24

### Pulmonary function results

With respect to PF tests at the final follow-up, we compared the PF test values of all 35 patients with the reference (predicted) values calculated by the spirometer. While there was a significant decrease in FVC and FEV_1_ of the patients at the final follow-up when compared with the reference values (p < 0.001 and p < 0.001, respectively), the FEV_1_/ FVC values at the final follow-up were not significantly different from the reference values (p = 0.25) (Table 2).

**Table 2:** Pulmonary function test of the 35 patients at final follow-up (paired t test).

	Absolute Value	Reference Value	P value	% Predicted
FVC (L)	2.5 ± 0.6 (1.0–3.9)	3.3 ± 0.4 (2.6–4.7)	< 0.001	76.4 ± 16.0 (40–119)
FEV_1_ (L)	2.2 ± 0.5 (1.3–3.4)	2.9 ± 0.3 (2.0–4.0)	< 0.001	82.8 ± 12.5 (44–104)
FEV_1_ / FVC	0.89 ± 0.1 (0.72–1.22)	0.87 ± 0.1 (0.70–1.21)	0.25	

FVC, forced vital capacity; FEV_1_, forced expiratory volume in 1 second.

The values are given as the average and the standard deviation.

Absolute and percent-predicted values pre and postoperatively at the final follow-up were analysed in all 35 patients (Table 3). Both FVC and FEV_1_ absolute values at the final postoperative follow-up were higher than the preoperative values; however, the differences were not statistically significant (p = 0.22 and p = 0.08, respectively). Percent-predicted FVC and FEV_1_ values at the pre and final postoperative follow-up showed no statistical difference (p = 0.63 and p = 0.29, respectively). The pre and final postoperative follow-up FEV_1_/ FVC values also showed no statistical difference (p = 0.14). However, for the patients who presented with pulmonary impairment preoperatively, both the FVC and FEV_1_ significantly increased at the final follow-up (Table 4).

**Table 3:** Comparison between preoperative and final follow-up pulmonary function test value of the 35 patients at final follow-up (paired t test).

	Preoperative	Final follow-up	P value
FVC (L)	2.3 ± 0.7 (0.6–3.8)	2.5 ± 0.6 (1.0–3.9)	0.22
Predicted FVC (L)	3.1 ± 0.5 (2.2–4.2)	3.3 ± 0.4 (2.6–4.7)	0.09
% FVC	74.0 ± 19.8 (15–114)	76.4 ± 16.0 (40–119)	0.63
FEV_1_ (L)	2.0 ± 0.6 (0.6–3.5)	2.2 ± 0.5 (1.3–3.4)	0.08
Predicted FEV_1_ (L)	2.5 ± 1.0 (1.2–4.0)	2.9 ± 0.3 (2.0–4.0)	0.06
% FEV_1_	91.8 ± 42.6 (15.4–203)	82.8 ± 12.5 (44–104)	0.29
FEV_1_ / FVC	0.86 ± 0.1 (0.59–1.02)	0.89 ± 0.1 (0.72–1.22)	0.14

FVC, forced vital capacity; FEV_1_, forced expiratory volume in 1 second.

The values are given as the average and the standard deviation.

**Table 4:** Comparison between preoperative and final follow-up pulmonary function test value of the 20 patients who had preoperative pulmonary impairment (paired t test).

	Preoperative	Final follow-up	p value
FVC (L)	2.1 ± 0.6 (0.6–3.2)	2.3 ± 0.5 (1.0–9.9)	0.01
Predicted FVC (L)	3.1 ± 0.5 (2.4–4.2)	3.3 ± 0.3 (2.6–3.8)	0.06
% FVC	66.1 ± 15.6 (15–79)	70.7 ± 13.2 (40–92)	0.29
FEV_1_ (L)	1.8 ± 0.5 (0.6–2.5)	2.1 ± 0.4 (1.3–2.6)	0.01
Predicted FEV_1_ (L)	2.6 ± 1.0 (1.2–4.1)	2.8 ± 0.3 (2.0–3.4)	0.12
% FEV_1_	79.2 ± 37.7 (15.4–169)	81.1 ± 9.1 (59–93)	0.60
FEV_1_ / FVC	0.87 ± 0.1 (0.59–1.02)	0.91 ± 0.1 (0.73–1.22)	0.22

FVC, forced vital capacity; FEV_1_, forced expiratory volume in 1 second.

The values are given as the average and the standard deviation.

Finally, preoperative PF tests of the 35 patients with the available preoperative data were compared with PF tests of those patients who refused to come to the final follow-up (n = 15), to analyse the representation of the study sample [4]. There were no significant differences in the preoperative PF test values between the 35 patients followed-up and the 15 patients without follow-up (p > 0.05) (Table 5).

**Table 5:** Comparison between preoperative pulmonary function test value of the 35 patients with follow-up and the 15 patients with basal value but without follow-up (Mann-Whitney *U* test).

	35 Patients Followed	15 Patients Only Baseline	P value
FVC (L)	2.3 ± 0.7 (0.6–3.8)	2.6 ± 0.7 (1.6–4.0)	0.23
Predicted FVC (L)	3.1 ± 0.5 (2.2–4.2)	3.5 ± 0.6 (2.6–4.7)	0.06
% FVC	76.3 ± 16.2 (37.3–114.1)	75.0 ± 11.9 (48.0–88.3)	0.80
FEV_1_ (L)	2.0 ± 0.6 (0.62–3.5)	2.2 ± 0.5 (1.4–3.0)	0.10
Predicted FEV_1_(L)	2.5 ± 1.0 (1.2–4.0)	3.0 ± 0.9 (1.2–3.9)	0.10
% FEV_1_	91.8 ± 42.6 (15.4–203.0)	79.8 ± 28.9 (44.0–162.3)	0.12

FVC, forced vital capacity; FEV_1_, forced expiratory volume in 1 second.

The values are given as the average and the standard deviation.

### Radiographic results

The average preoperative MT curve of the 35 studied patients was 68.1° (42–122°), average preoperative PT curve was 34.6° (9–85°), and average preoperative TL/L curve was 45.7° (14–82°). Sagittal plane analysis revealed that the average preoperative thoracic kyphosis was 16.2° (4–38°), and lumbar lordosis was 39.4° (20–56°). With regard to the coronal and sagittal balance, the average preoperative C7 from CSVL was 15.1 mm (0–35 mm) and from sagittal vertical axis was 17.5 mm (-28–65 mm). When compared with the 15 patients who were not included in the study (dropped out), statistical differences were not observed between the preoperative values of the two groups (p > 0.05). Table 6 summarises this comparison as well as the postoperative and final follow-up radiographic outcomes of the studied patients [4].

**Table 6:** Radiographic parameters of the studied patients compared with the dropped out patients (Mann-Whitney *U* test).

	Dropped Out Patients (n = 15)	Studied Patients (n = 35)			Dropped Out versus Studied Patients
	Preoperative	Preoperative	Postoperative	Final Follow-up	P value
Proximal thoracic curve (º)	27.1 ± 6.7 (17–35)	34.6 ± 20.3 (9–85)	17.5 ± 10.4 (5–52)	23.0 ± 5.8 (17–34)	0.47
Main thoracic curve (º)	58.3 ± 5.1 (50–69)	68.1 ± 18.6 (42–122)	23.5 ± 2.0 (8–71)	28.9 ± 2.2 (10–72)	0.09
Thoracolumbar/Lumbar curve (º)	36.8 ± 12.5 (17–55)	45.7 ± 14.4 (14–82)	22.1 ± 11.2 (4–46)	20.0 ± 10.2 (4–43)	0.06
Thoracic kyphosis (T5 to T12) (º)	17.1 ± 11.2 (-7–29)	16.2 ± 8.8 (4–38)	18.8 ± 9.0 (4–40)	21.3 ± 8.2 (5–42)	0.39
Lumbar lordosis (L1 to S1) (º)	40.5 ± 10.6 (25–54)	39.4 ± 11.1 (20–56)	34.4 ± 10.9 (15–60)	36.9 ± 9.2 (13–55)	0.84
C7 translation from central sacral vertical line (mm)	14 ± 5.4 (7–20)	15.1 ± 10.2 (0–35)	11.1 ± 7.5 (0–32)	13.5 ± 7.0 (0–30)	0.96
Sagittal vertical axis (mm)	19.6 ± 13.6 (5–35)	17.5 ± 25.5 (-28–65)	20.2 ± 22.7 (-40–60)	9.8 ± 28.1 (-43–62)	0.87

The values are given as the average and the standard deviation.

To determine if the lack of improvement in PF test values is due to the baseline deformity, the PF test values were correlated with the severity of the preoperative radiographic metrics. There were no significant correlations between the PF test values and preoperative radiographic metrics (Table 7). In addition, to determine if, although on average there was not a significant change from preoperative to postoperatively, the patients who had a greater radiographic correction also had a greater correction in PF test values, the amount of improvement of PF test values was correlated with the amount of improvement of the postoperative radiographic parameters. The results showed that there were no significant correlations between the degree of improvement in the PF test values and degree of improvement in the postoperative radiographic parameters (Table 8). Finally, to determine if there is a threshold radiographic correction above which a patient will experience an improvement in PF test values, the amount of improvement of the PF test values was correlated with the amount of the final postoperative radiographic metrics. The results showed that there were no significant correlations between the degree of improvement in the PF test values and degree of improvement in the final postoperative radiographic parameters (Table 9).

**Table 7:** Correlation analysis between pulmonary function test value and preoperative radiographic parameters.

	MT curve		PT curve		TL/L curve		Thoracic kyphosis		Lumbar lordosis		C7-CSVL		Sagittal vertical axis	
	r	p	r	p	r	p	r	p	r	p	r	p	r	p
FVC (L)	-0.42	0.06	-0.43	0.06	0.04	0.85	-0.19	0.41	-0.02	0.93	0.09	0.69	-0.27	0.25
%FVC	-0.37	0.11	-0.48	0.06	0.10	0.69	-0.10	0.67	-0.08	0.72	0.25	0.29	-0.38	0.10
FEV_1_(L)	-0.40	0.08	-0.39	0.09	-0.09	0.70	-0.27	0.25	-0.13	0.57	0.09	0.70	0.03	0.27
%FEV_1_	-0.03	0.89	-0.27	0.25	-0.16	0.50	-0.06	0.81	-0.27	0.24	0.33	0.15	0.03	0.27
FEV_1_/FVC	0.09	0.71	0.18	0.46	-0.34	0.15	-0.22	0.35	-0.34	0.14	0.04	0.86	0.07	0.91

MT, main thoracic; PT, proximal thoracic; TL/L, thoracolumbar/lumbar; CSVL, center sacral vertical line.

**Table 8:** Correlation analysis between change in pulmonary function test value and change in radiographic parameters.

	MT curve		PT curve		TL/L curve		Thoracic kyphosis		Lumbar lordosis		C7-CSVL		Sagittal vertical axis	
	r	p	r	p	r	p	r	p	r	p	r	p	r	p
FVC (L)	0.40	0.12	0.24	0.38	-0.32	0.22	-0.07	0.81	0.16	0.57	-0.03	0.91	0.40	0.12
%FVC	0.39	0.13	0.37	0.16	-0.34	0.20	-0.05	0.84	-0.12	0.66	-0.17	0.52	0.38	0.14
FEV_1_(L)	0.34	0.20	0.23	0.38	-0.26	0.34	-0.02	0.94	0.05	0.85	-0.29	0.27	0.22	0.41
%FEV_1_	0.01	0.97	0.21	0.44	-0.17	0.54	0.11	0.68	-0.11	0.68	-0.29	0.27	-0.12	0.65
FEV_1_/FVC	0.12	0.66	0.09	0.75	-0.10	0.69	0.12	0.66	-0.08	0.78	-0.45	0.08	-0.11	0.68

MT, main thoracic; PT, proximal thoracic; TL/L, thoracolumbar/lumbar; CSVL, center sacral vertical line.

**Table 9:** Correlation analysis between change in pulmonary function test value and postoperative radiographic parameters.

	MT curve		PT curve		TL/L curve		Thoracic kyphosis		Lumbar lordosis		C7-CSVL		Sagittal vertical axis	
	r	p	r	p	r	p	r	p	r	p	r	p	r	p
FVC (L)	0.24	0.31	0.32	0.17	0.28	0.23	0.07	0.76	0.35	0.13	0.24	0.32	0.21	0.38
%FVC	0.14	0.57	0.40	0.08	-0.04	0.85	0.13	0.58	0.06	0.81	0.16	0.49	-0.04	0.86
FEV_1_(L)	0.33	0.15	0.17	0.48	0.38	0.10	0.20	0.41	0.35	0.13	0.16	0.49	0.27	0.24
%FEV_1_	0.39	0.09	0.14	0.55	0.21	0.38	0.16	0.51	0.05	0.85	0.20	0.41	0.20	0.40
FEV_1_/FVC	0.38	0.10	-0.11	0.66	0.30	0.20	0.25	0.30	0.27	0.24	0.09	0.72	0.23	0.32

MT, main thoracic; PT, proximal thoracic; TL/L, thoracolumbar/lumbar; CSVL, center sacral vertical line.

## Discussion

This study aimed to investigate long-term PF after posterior spinal fusion for treating main thoracic adolescent idiopathic scoliosis and found that posterior spinal fusion did not decrease PF 15 years after the initial surgery.

While new alveoli develop until the age of 5 to 8 years [4, 5, 14], PF and respiratory maturation proceeds until the age of 17 to 23 years [4, 5, 15]. Because thoracic AIS patients may demonstrate a restrictive pulmonary impairment and PF may correlate with the degree of scoliosis [4, 16], it is necessary to investigate whether surgical interventions affect postoperative PF. In this study, the mean age at the final follow-up was 34 years, suggesting that we analysed the PF at the optimal time because PF gradually decreases after the age of 35 years [5].

Few studies have reported the long-term (>10 years) PF after a posterior approach [5, 9, 10]. In a 10-year follow-up analysis, Gitelman et al [5]. firstly reported that AIS patients that underwent posterior spinal fusion/instrumentation and no chest cage disruption experienced a significant increase in both FVC and FEV_1_, but no changes in percent-predicted values from baseline to 10 years. However, the major limitation of the study was that only approximately 7% of eligible patients returned for the long-term follow-up and only seven patients with MT curve were included [5]. Although the returning rate was not reported, Nohara et al [9]. documented that PF test results improved in 30 patients with thoracic AIS (Lenke 1 or 2) who underwent posterior spinal fusion with hybrid construct. Preoperative FVC and percent-predicted FEV_1_ was significantly improved at an average 14-year follow-up (2.5 L versus 2.7 L, 84% versus 87%, respectively). Akazawa et al [10]. reported that 18 AIS patients treated with spinal fusion with Harrington instrument showed restrictive ventilation defects in 27.7%. Although the mean duration of follow-up in their study was over 35 years, preoperative PF test data were unavailable and returning rate was only 9.2% [10].

In this study, the average percent-predicted FVC and FEV_1_/ FVC values at the final follow-up were 76.4% and 0.89, respectively, suggesting mild restrictive pulmonary impairment but no airflow obstruction. Considering the preoperative percent-predicted FVC value (74.0%), the restrictive pulmonary impairment—associated with thoracic AIS—existed before surgery. In addition, when comparing the change in the absolute and percent-predicted PF test values from before surgery to the final follow-up, there was no significant differences between pre and final postoperative values. In patients who presented with mild pulmonary impairment preoperatively, both the FVC and FEV_1_ significantly increased at the final follow-up. These results indicate that posterior spinal fusion did not decrease PF in AIS patients with MT curves in the long-term (>10 years). Although the patients with preoperative pulmonary impairment showed some significant improvements in the PF test values, there was no significant correlation between the PF test values and radiographic metrics. These results indicate that the lack of improvement is not due to baseline deformity. In addition, the results suggest that the patients who showed greater radiographic correction did not show correction of the PF test values. There is no radiographic correction threshold above which a patient will present with an improvement in the PF test results. Hence, it is possible that the correction, while showing improvement compared to the baseline value, was not good enough to improve the PF. Alternatively, it is possible that patients may not improve because alveolar underdevelopment during the preoperative period limits the extent to which the lungs can return to the normal state during the postoperative period. It is also possible that the measured PF metrics are not sensitive enough to detect a change.

Sudo et al [3]. reviewed 30 consecutive cases of Lenke type 5C major TL/L curves treated with anterior major curve correction (returning rate was 100%). With a mean follow-up duration of 17 years and mean follow-up age of 32 years, the mean preoperative FVC (2.7 L) and FEV (2.3 L) were not significantly different from the long-term follow-up measurements (2.8 L and 2.4 L, respectively) [3]. Similarly, the mean preoperative percent-predicted FVC (94%) and FEV_1_ (82%) were not significantly different from the long-term follow-up measurements (92% and 82%, respectively) [3]. Ruiz-Juretschke et al [4]. also documented that no major pulmonary impairment was observed in their 24 TL/L AIS patients who underwent open anterior surgery and were followed-up at 18 years (returning rate was 34%). In addition, Sudo et al [7]. assessed the long-term PF after open anterior spinal fusion for treating consecutive 25 thoracic AIS patients (returning rate was 100%). With a mean follow-up duration of 15 years and the mean age at the final follow-up of 32 years, the mean preoperative FVC (2.4 L) and FEV (2.1 L) were not significantly different from the long-term follow-up measurements (2.4 L and 2.1 L, respectively) [7]. However, the mean preoperative percent-predicted FVC (81%) and FEV_1_ (80%) were significantly decreased at the long-term follow-up (73% and 69%, respectively) [7]. These results were also supported by an international bi-centre long-term follow-up (14 years) study with 41 thoracic AIS patients (returning rate was 100%), showing that the preoperative percent-predicted FVC dropped from 80% to 72% at the final follow-up [8]. These findings suggest that among the several surgical approaches for AIS patients, the anterior approach for thoracic AIS is the most likely to affect the PF in the long-term.

The strength of the present study is that the mean follow-up period was over 15 years after surgery, making this study the largest regarding the number of patients and with the longest followed series described in the literature. In addition, the returning rate was relatively high (70%). Because of similar (not statistically different) baseline PF test values and radiographic parameters for all of the variables, the current study group was defined as a representative of the whole group of patients who underwent surgery by the posterior approach.

This study has a few limitations. First, the surgical procedure in the present study was segmental pedicle screw in a lumbar lesion and hook and segmental wiring in a thoracic lesion. The long-term effects on PF caused by modern posterior spinal fusion with all pedicle screw instrumentation remain unclear. Second, the current study only evaluated the effect of the surgical approach on PF. Other clinical outcomes such as pain, self-image, or satisfaction with management were not considered. Last, although there were no significant differences in the predicted FVC and TL/L curve between the 35 patients followed-up and the 15 patients without follow-up, the p-value was not marginally significant. This represents a potential source of bias in the study.

## Conclusions

The long-term results of AIS patients who underwent posterior spinal fusion in MT curves demonstrated absolute and percent-predicted PF test values similar to preoperative measurements; thus, indicating that posterior spinal fusion did not decrease PF 15 years after the initial surgery.
